# Influence of tunnel ionization to third-harmonic generation of infrared femtosecond laser pulses in air

**DOI:** 10.1038/s41598-020-74263-x

**Published:** 2020-10-15

**Authors:** Viktorija Tamulienė, Greta Juškevičiūtė, Danas Buožius, Virgilijus Vaičaitis, Ihar Babushkin, Uwe Morgner

**Affiliations:** 1 Laser Research Center, Vilnius University, Saulėtekio 10, 10223 Vilnius, Lithuania; 2 Institute of Quantum Optics, Leibniz University of Hannover, Hannover, Germany; 3 Max Born Institute, Max-Born-Straße 2A, 12489 Berlin, Germany; 4 Cluster of Excellence PhoenixD (Photonics, Optics, and Engineering-Innovation Across Disciplines), Welfengarten 1, 30167 Hannover, Germany

**Keywords:** Optics and photonics, Optical physics

## Abstract

Here we present an experimental as well as theoretical study of third-harmonic generation in tightly focused femtosecond filaments in air at the wavelength of $1.5 \,\upmu \hbox {m}$. At low intensities, longitudinal phase matching is dominating in the formation of 3rd harmonics, whereas at higher intensities locked X-waves are formed. We provide the arguments that the X-wave formation is governed mainly by the tunnel-like ionization dynamics rather than by the multiphoton one. Despite of this fact, the impact of the ionization-induced nonlinearity is lower than the one from bound–bound transitions at all intensities.

## Introduction

Third harmonic generation (THG) and laser frequency tripling is one of the most important nonlinear optical phenomena. Its application areas range from development of coherent radiation sources in ultraviolet (UV) and vacuum UV (VUV) ranges^1,2^, nonlinear media and plasma characterization^3,4^, nonlinear spectroscopy and microscopy^5,6^ to temporal laser pulse characterization^7,8^, white light filament formation and remote atmosphere sensing^9,10^.

THG in air is accompanied by plasma generation and filament formation. Kerr self-focusing leads to a beam collapse which is arrested by plasma defocusing and nonlinear losses^11^. An important part of this filament formation picture is a description of ionization process which is very often made in the terms of multiphoton dynamics. In multiphoton ionization regime, as contrasted to tunneling one, ionization takes place on slow time scale comparable to the pulse envelope and ionization rate grows as some power of intensity, whereas in the tunneling regime the dynamics inside the laser cycle plays the key role, and ionization is described as an exponential function of instantaneous intensity. The key quantity defining which of these two seemingly very different regimes plays a role, is so called Keldysh parameter $\gamma = 2\pi \tau _I/T$, describing the characteristic time at which the ionization occurs $\tau _I = \sqrt{2I_P}/E$, in relation to a single optical cycle *T* (here the expression for $\tau _I$ is written in atomic units, $I_P$ is the ionization potential and *E* is the electric field). The multiphoton range takes place for $\gamma \gg 1$, whereas for $\gamma \ll 1$ the tunnel ionization is more appropriate description. For the filaments obtained from femtosecond Ti:Sapphire laser pulses with the central wavelength of about 800 nm, intensity clamping takes place at intensities of the order of $10^{13}$ W/cm$^2$, which corresponds to $\gamma \approx 2$, at the boundary of the multiphoton range. At longer pump wavelengths, one can approach this boundary ($\gamma =1$) or even cross it^12^. Such long wavelength filaments can be obtained using pump from high power optical parametric amplifiers, and are actively studied nowadays.

One of the major difficulties arising in the intermediate region $\gamma \approx 1$ is that neither tunnel nor multiphoton formulas work well. Nevertheless, there are few ionization models which are able to fill the gap^13,14^. In particular, in the work of Yudin and Ivanov^14^ an approach was developed which allowed to describe not only the scaling of ionization with the driving field intensity correctly in tunnel, multiphoton and transient regions, but also both the fast intra-cycle tunnel-like and slow intercycle multiphoton-like dynamics of ionization.

In the tunnel regime, ionization-based nonlinearity creates also harmonics of the fundamental, which are often called Brunel harmonics^15,16^. In the multiphoton regime, the Brunel harmonics are not generated, because in this case the ionization nonlinearity is “too slow” with respect to the cycle period of the third harmonic radiation^15^ . Ionization-based radiation mechanism is related to transitions of electrons which were bounded inside the atom before the pulse, but remain free after it. This is in contrast to transitions giving impact into $\chi ^{(3)}$ nonlinearity, corresponding to electrons which remain bound before, after, and during the pulse. To mention here is also the transitions resulting in so called high-harmonic generation (HHG), which start from bound state and end there, but the electron spends some time outside the atom.

Third harmonic generated in filaments has impacts from the $\chi ^{(3)}$-based nonlinearity as well as from ionization-based nonlinearity. Theoretical estimations^17^ show that, whereas for many gases at 800 nm wavelength $\chi ^{(3)}$-based mechanism should dominate, situation changes for longer wavelengths. In particular, in experimental work^16^ arguments were provided, that at the mid-infrared wavelengths around $4\, \upmu \hbox {m}$ Brunel mechanism dominates for several harmonics, presumingly including the third one.

In general, there are number of works which study THG induced by infrared (IR) laser pulses^1,3–5,9,18–24^. One of the important pecularities by filament propagation is a generation of so-called X-waves. An X-wave is a multicolor conical wave with each cone travelling at the same group velocity. Such X-waves were first suggested to appear in the fundamental harmonic and are explained by spatio-temporal reshaping governed by dynamical nonlinear phase-matched four-wave mixing processes^25,26^. Later on, such characteristic X-shaped distributions were observed in the third harmonic^22–24^.

In this report, we present results of experimental and theoretical investigation of THG in air by IR femtosecond laser pulses at 1.5 $\upmu$m. Theoretically, we approached the process using two models: the multiphoton one^27^, which included only slow inter-cycle ionization and the nonadiabatic tunnel-based Yudin–Ivanov one^14^, demonstrating sub-cycle ionization features (see above). Comparison of the results of the two models with experiment shows that the tunnel-based model explains the observed experimental data much better. In particular, the tunnel-based model predicts higher clamping intensities, stronger influence of ionization on the dispersion of the fundamental and third harmonics and, as a result, a stronger divergence of X-waves. Moreover, in the Yudin–Ivanov model we compared the impacts of the bound–bound and $\chi ^{(3)}$ nonlinearities to the third harmonic.

## Methods

### Experimental set-up

For the experiments we have used a 1 kHz repetition rate femtosecond Ti:sapphire chirped pulse amplification laser system (Legend elite duo HE+, Coherent Inc.), delivering 35–40 fs (FWHM) light pulses centred at 790 nm with maximal pulse energy of 8 mJ. The laser output was used to pump an optical parametric amplifier (OPA, Light Conversion, Inc.), which produced the tunable infrared (IR) signal and idler wave pulses in the wavelength range between 1.2 and 1.6 $\upmu$m and 1.6 and 2.4 $\upmu$m, respectively. The duration and single-pulse energy of the IR signal wave pulses was about 40 fs and 1.3–1.75 mJ, respectively. The beam width at FWHM was about 4 mm. The IR signal wave pulses were focused in air by the lenses of various focal lengths, which resulted in a visible third harmonic generation.

**Figure 1:**
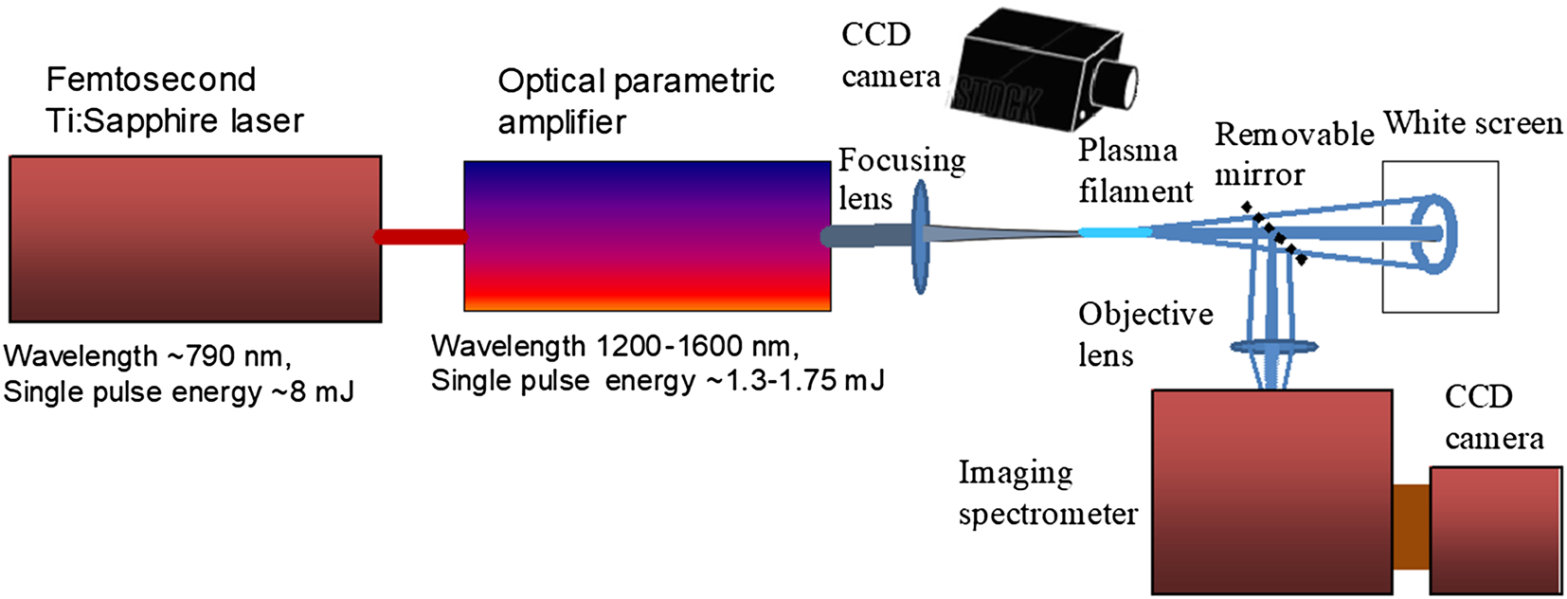
Experimental set-up.

Far-field patterns of third harmonic radiation were observed on a white screen placed at about 1 m beyond the focal plane of the focusing lens and registered by a CCD camera (Fig. 1). The spectra of this radiation have been recorded with a help of a fibre spectrometer sensitive in the visible and IR spectral ranges. Frequency-angular distributions of third harmonic pulses were recorded with an imaging spectrometer Andor SR-500i-D1 by placing its entrance slit in the focal plane of an objective lens of the short focal length (10–20 cm). Under such lens and slit configuration the spectrometer disperses the light energy across a wide spectral range and produces a two-dimensional image at the output imaging plane (the vertical coordinate is proportional to the light direction). Assuming the axial symmetry of the emissions and by placing the CCD camera in the output imaging plane of the spectrometer, the frequency-angular distributions can be captured in a single shot.

### Numerical modelling of THG in air

Here, we describe two mathematical models of THG in air, which we call “MPI model” and “YI model”. The MPI model is based on the slow varying envelope approximation (SVEA) for the field, and accounts only the multiphoton ionization of air, neglecting the tunnel ionization. In contrast, the YI model is free from SVEA, that is, formulated directly for the fast optical electric field, and uses the formula of Yudin and Ivanov (YI)^14^, which takes into account both slow multiphoton and fast tunnel components of the ionization dynamics. In the MPI model, plasma density depends on the light intensity and the Brunel harmonics are not generated. Third harmonic generation is predefined only by the third-order Kerr term. Because of SVEA, two separate governing equations for the fundamental harmonic (FH) and third harmonic (TH) are used. On the other hand, the YI model allows generation of the third harmonic also via plasma-based nonlinearity. In both models, so called slowly evolving wave^28^ and paraxial approximations were used.

**Figure 2:**
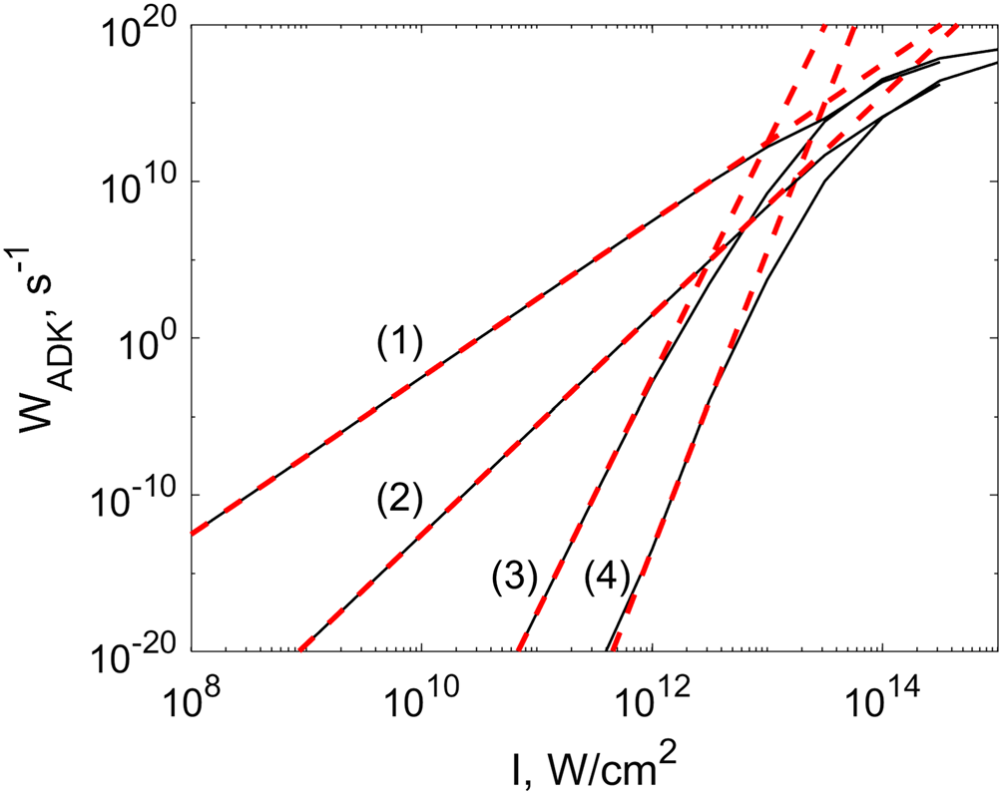
Intensity dependence of ionization rate. Black solid lines: ADK formula^27^, red dashed lines: linear approximation with parameters of Table 1. Oxygen (1,3) and nitrogen (2,4); third (1,2) and fundamental (3,4) harmonics. At high intensities, linear approximation overestimates the plasma density.

#### MPI model

In the MPI model, we solve the following governing equations for the fundamental harmonic (FH) and third harmonic (TH) waves:

1 $$\begin{aligned} \frac{\partial \widehat{\mathcal {E}}_1}{\partial z}=ik_{1z}\widehat{\mathcal {E}}_1+\widehat{P}_1^{(3)}+\widehat{P}_1^{(pl)},\end{aligned}$$

2 $$\begin{aligned} \frac{\partial \widehat{\mathcal {E}}_3}{\partial z}=ik_{3z}\widehat{\mathcal {E}}_3+\widehat{P}_3^{(3)}+\widehat{P}_3^{(pl)}, \end{aligned}$$

where $\widehat{\mathcal {E}}_{1,3}(\Omega ,\beta _x,\beta _y,z)$ stands for the Fourier transform of the complex slow envelopes $\mathcal {E}_{1,3}(t,x,y,z)$ of the FH (index 1) and TH (index 3) of the fast optical field *E*(*t*, *x*, *y*, *z*). Here *z* is the propagation distance, *x* and *y* are the transverse coordinates and *t* is time. $\Omega =\omega -\omega _{j0}$, where $\omega _{j0}=2\pi c/\lambda _{j0}$ is the central cyclic frequency of the *j*th wave ($j=1,3$) and $\omega$ is the cyclic frequency. $\lambda _{j0}$ is the central wavelength and *c* is the speed of light.

The first rhs term in Eqs. (1) and (2) describes free-space propagation in air. $k_{1z}$ and $k_{3z}$ are the *z*-projections of the wavevectors centered at the corresponding frequencies, e.g. $k_{1,3z}(\Omega )=k(\omega _{1,30}+\Omega )-(\beta _x^2+\beta _y^2)/(2k_{1,30})-\Omega /u_{10}$ and $u_{10}$ is the group velocity of the fundamental wave in air. Here, $k(\omega )=\omega n(\lambda )/c$, $\lambda =2\pi c/\omega$ is the wavelength, *n* is the wavelength-dependent refractive index determined by the Sellmeier equations for air^29^, and $k_{1,30}$ is the wavenumber at the central frequency $\omega _{1,30}$. The shift $\Omega /u_{10}$ is introduced to consider both harmonics in the reference frame of the FH pulse.

The nonlinear terms are given by:

3 $$\begin{aligned} \widehat{P}_1^{(3)}= & {} (\omega _{10}+\Omega )\left( i\widehat{P}_{Kerr,1}+i\widehat{P}^{(a)}\right) ,\end{aligned}$$

4 $$\begin{aligned} \widehat{P}_3^{(3)}= & {} (\omega _{30}+\Omega )\left( i\widehat{P}_{Kerr,3}+i\widehat{P}^{(b)}\right) . \end{aligned}$$

The first term, $\widehat{P}_{Kerr}$, includes self- and cross-phase modulations. It is the Fourier transform of the following expressions:

5 $$\begin{aligned} P_{Kerr,1}=\frac{1}{c}n_2\left( I_1+2I_3\right) {\mathcal {E}}_1,\end{aligned}$$

6 $$\begin{aligned} P_{Kerr,3}=\frac{1}{c}n_2\left( 2I_1+I_3\right) {\mathcal {E}}_3, \end{aligned}$$

where $n_2$ is the nonlinear refractive index of air, $I_j=c\varepsilon _0|\mathcal {E}_j|^2/2$ is the intensity ($j=1,3$), $\varepsilon _0$ is the vacuum permittivity.

$\widehat{P}^{(a)}$ and $\widehat{P}^{(b)}$ are the Fourier transforms of $\varepsilon _0n_2{\mathcal {E}}_3{\mathcal {E}}_1^{*2}/2$ and $\varepsilon _0 n_2{\mathcal {E}}_1^{3}/6$, respectively. The last term generates the third harmonic due to the $\chi ^{(3)}$ nonlinearity.

The plasma terms $\widehat{P}^{(pl)}_{1,3}$ are related to the plasma density described by the equation:

7 $$\begin{aligned} \frac{\partial \rho ^{(n)}}{\partial t}=[1-\rho ^{(n)}(t)]W(t), \end{aligned}$$

where $\rho ^{(n)}$ is the normalized to the neutral density $\rho _0$ plasma density and *W*(*t*) is the generation rate. In MPI model,

8 $$\begin{aligned} W(t)=\sum \limits _{j=1,3}[0.2\Gamma _{jO}I^{K_{jO}}+0.8\Gamma _{jN}I^{K_{jN}}]. \end{aligned}$$

The plasma is generated both by FH ($j=1$) and TH ($j=3$). Two main constituents of air–oxygen (O) and nitrogen (N)—are taken into account. Coefficients $\Gamma$ were calculated using the ADK-formula taken from^27^ (in^27^, the misprints were corrected). The intensity dependence of the ionization rate was fitted by a line in the logarithmic scale and $\Gamma$ was found, Fig. 2. The linear approximation was performed with the formula $\log _{10}(W_{ADK})=\log _{10}(\Gamma )+K\log _{10}(I)$. *K* is the photon number needed for the multiphoton ionization. The coefficients are given in Table 1.

**Table 1:** Parameters of the MPI model at wavelengths $\lambda _{10}=1.5$ $\upmu$m and $\lambda _{30}=0.5$ $\upmu$m.

Parameter	Value	Units
$K_{1O}$	15	
$K_{3O}$	5	
$\Gamma _{1O}$	$10^{-242.6}$	(m$^2$/W)$^{15}$s$^{-1}$
$\Gamma _{3O}$	$10^{-72.5}$	(m$^2$/W)$^5$s$^{-1}$
$K_{1N}$	19	
$K_{3N}$	7	
$\Gamma _{1N}$	$10^{-317.5}$	(m$^2$/W)$^{19}$s$^{-1}$
$\Gamma _{3N}$	$10^{-110.6}$	(m$^2$/W)$^{7}$s$^{-1}$

In Eqs. (1) and (2) the plasma terms read:

9 $$\begin{aligned} \widehat{P}_{1}^{(pl)}=\frac{-iq_e^2}{2m_e c\varepsilon _0}FT\left[ \rho \mathcal {E}_1\right] /(\omega _{10}+\Omega )-J_{loss,1}, \end{aligned}$$

10 $$\begin{aligned} \widehat{P}_{3}^{(pl)}=\frac{-iq_e^2}{2m_e c\varepsilon _0}FT\left[ \rho \mathcal {E}_3\right] /(\omega _{30}+\Omega )-J_{loss,3}, \end{aligned}$$

where $FT[\circ ]$ denotes the Fourier transform, $q_e$ and $m_e$ are the electron charge and mass, respectively. The loss terms for the multiphoton ionization are given by

11 $$\begin{aligned} J_{loss,1}=FT\left[ \frac{1}{2}\mathcal {E}_1\rho _0\left( 0.2K_{1O}\hslash \omega _{10}\Gamma _{1O}I_1^{K_{1O}-1}+0.8K_{1N}\hslash \omega _{10}\Gamma _{1N}I_1^{K_{1N}-1}\right) \right] ,\end{aligned}$$

12 $$\begin{aligned} J_{loss,3}=FT\left[ \frac{1}{2}\mathcal {E}_3\rho _0\left( 0.2K_{3O}\hslash \omega _{30}\Gamma _{3O}I_3^{K_{3O}-1} +0.8K_{3N}\hslash \omega _{30}\Gamma _{3N}I_3^{K_{3N}-1}\right) \right] . \end{aligned}$$

#### YI model

In this model, we solve a single governing equation for the Fourier transform $\widehat{\mathcal {E}}(\omega ,\beta _x,\beta _y,z)$ of the analytical signal $\mathcal {E}(t,x,y,z)$ (the real optical electric field is related to $\mathcal {E}$ as $E(t,x,y,z) = \mathfrak {R}( \mathcal {E}(t,x,y,z))$, where $\mathfrak {R}(\circ )$ denotes the real part):

13 $$\begin{aligned} \frac{\partial \widehat{\mathcal {E}}}{\partial z}=ik_{z}\widehat{\mathcal {E}}+\widehat{P}^{(3)}+\widehat{P}^{(pl)}. \end{aligned}$$

The fundamental and third harmonics are extracted from $\widehat{\mathcal {E}}$ by truncating the spectrum in the following intervals: $0.5<\omega /\omega _{10}<1.5$ (FH frequency interval) and $2.5<\omega /\omega _{10}<3.75$ (TH frequency range). In Eq. (13), the linear dispersion is described by $k_{z}=k(\omega )-(\beta _x^2+\beta _y^2)/(2Qk_{10})-\omega /u_{10}$, where $Q=3$ for the TH frequency interval and $Q=1$ elsewhere.

The Kerr nonlinear term is calculated as:

14 $$\begin{aligned} \widehat{P}^{(3)}=i\omega \widehat{P}_{K}, \end{aligned}$$

where $\widehat{P}_K$ is the Fourier transform of $P_K=4n_2\varepsilon _0 [\mathfrak {R}({\mathcal {E}})]^3/3$.

Plasma generation is described by Eq. (7). Here, for the tunnel ionization rate, the Yudin–Ivanov^14^ formula (in atomic units) for the instantaneous photoemission rate was used:

15 $$\begin{aligned} W(\omega t)=\frac{\pi }{\tau _{T}}\exp \left( -\sigma _{0}\frac{|\mathcal {E}|^{2}}{\omega ^{3}}\right) \left[ \frac{2\kappa ^{3}}{|\mathcal {E}|}\right] ^{2Z/\kappa } \exp \left[ -\frac{|\mathcal {E}|^{2}}{2\omega ^{3}}\sigma _{1}\sin ^{2}(\mathrm {arg}(\mathcal E))\right] , \end{aligned}$$

where *Z* is the atomic charge, $\kappa =\sqrt{2\phi }$, $\phi$ is the ionization potential (in atomic units), $\tau _{T}=\kappa /|\mathcal {E}|$, $\sigma _{0}=0.5(\gamma ^{2}+0.5)\ln C-0.5\gamma \sqrt{1+\gamma ^{2}}$, $\gamma =\omega \tau _{T}$ is the Keldysh parameter, $C=1+2\gamma \sqrt{1+\gamma ^{2}}+2\gamma ^{2}$ and $\sigma _{1}=\ln C$-$2\gamma /\sqrt{1+\gamma ^{2}}$. In Eq. (15), we used the fundamental harmonics $\mathcal {E}_1$ in place of $\mathcal {E}$ since YI formula was derived for a single-color field. $\mathcal {E}_1$ is obtained by truncating the spectral amplitude $\widehat{\mathcal {E}}$ to the frequency interval of the fundamental harmonic as defined after Eq. (13).

For the plasma term, the following formula is utilized:

16 $$\begin{aligned} P^{(pl)}=\frac{-iq_e^2}{2m_e c\varepsilon _0}FT\left[ \rho \mathcal {E}_1\right] /(\omega +i\gamma _r)-J_{loss}, \end{aligned}$$

where^30^ $\gamma _r=1/200$ fs$^{-1}$ and

17 $$\begin{aligned} J_{loss}=FT\left[ \left( 0.2W_O(t)U_{O}+0.8W_N(t)U_N\right) \frac{(\rho _0-\rho )}{\mathfrak {R}(\mathcal {E}_1)2\varepsilon _0 c} \right] . \end{aligned}$$

$U_O$ and $U_N$ are the ionization energies of oxygen and nitrogen, respectively. These ionization potentials are also utilized when calculating $W_{O,N}(t)$.

**Figure 3:**
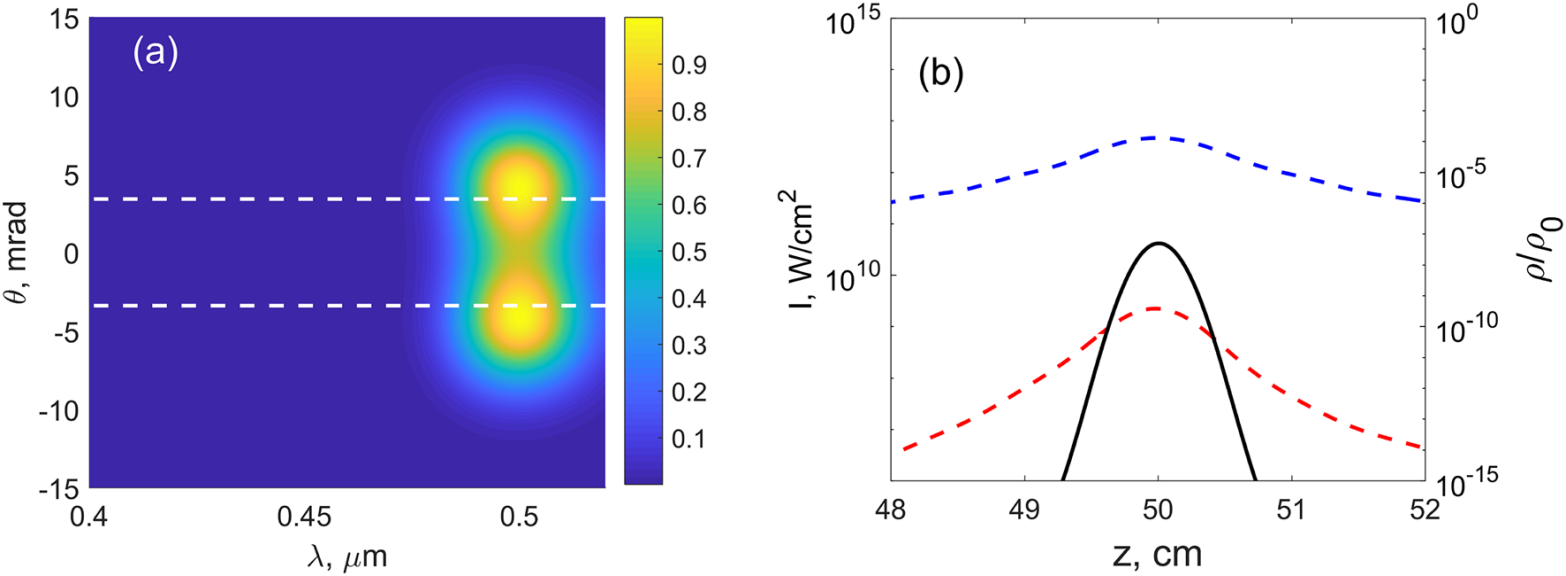
Numerical results for the MPI model at low input intensities. (**a**) Output TH spectrum. (**b**) Dependencies of the FH (blue dashed line) and TH (red dashed line) intensities as well as plasma density (black solid line) on the propagation distance (here and further the propagation only near the focal region is shown). $f=50$ cm, $I_0=0.044\times 10^{10}$ W/cm$^2$. Dashed lines in (**a**) denote the longitudinal phase-matching angle (see text).

#### The input FH beam

We assume no TH at the input and the FH is focused by a lens with the focus length *f* placed at $z=0$. Then, at $z=0$, the initial conditions read:

18 $$\begin{aligned} \mathcal {E}_1(t,x,y,z=0)= & {} E_0 \exp \left( -(x^2+y^2)\left[ \frac{1}{r_0^2}+\frac{i\pi }{\lambda _{10}f}\right] \right) \exp \left( -\frac{t^2}{\tau ^2}\right) ,\end{aligned}$$

19 $$\begin{aligned} \mathcal {E}_3(t,x,y,z=0)= & {} 0 \end{aligned}$$

for the MPI model, and $\mathcal {E}(t,x,y,z=0)=\mathcal {E}_1(t,x,y,z=0)e^{-i\omega _{10}t}$ for the YI model. Here, $r_0$ is the beam radius and $\tau$ is the pulse duration. $E_0$ is the peak amplitude and the corresponding input peak intensity is noted as $I_0$.

## Results

**Figure 4:**
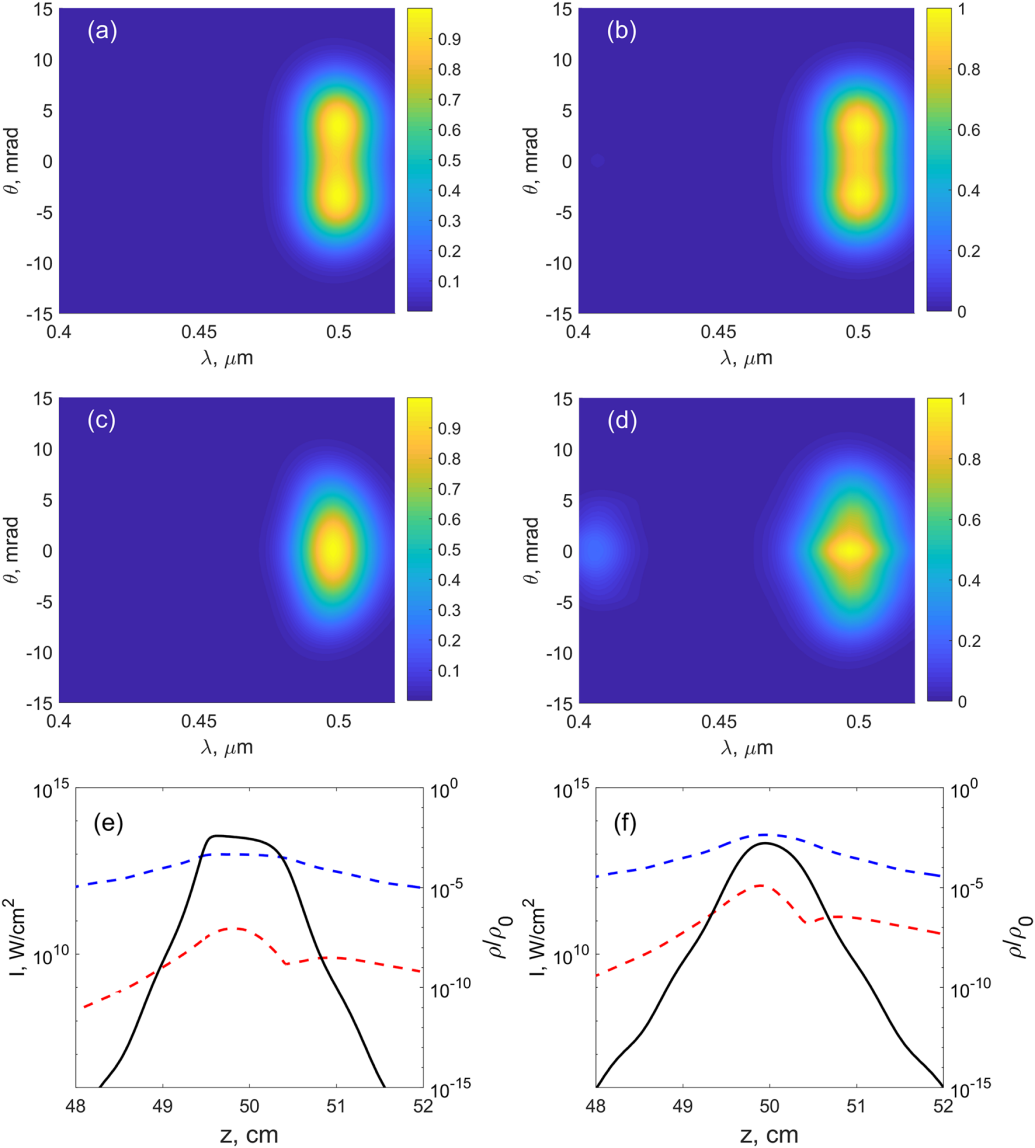
Numerical results for the MPI model (**a**,**c**,**e**) and YI model (**b**,**d**,**f**) for higher input intensities. (**a**–**d**) Output TH spectra. (**e**,**f**) Dependencies of the FH (blue dashed line) and TH (red dashed line) intensities as well as plasma density (black solid line) on the propagation distance. (Here and in other figures the peak intensity is shown.) $f=50$ cm, $I_0$ [$10^{10}$ W/cm$^2$]: 0.088 (**a**), 0.175 (**c**,**e**); 0.175 (**b**), 0.35 (**d**,**f**).

**Figure 5:**
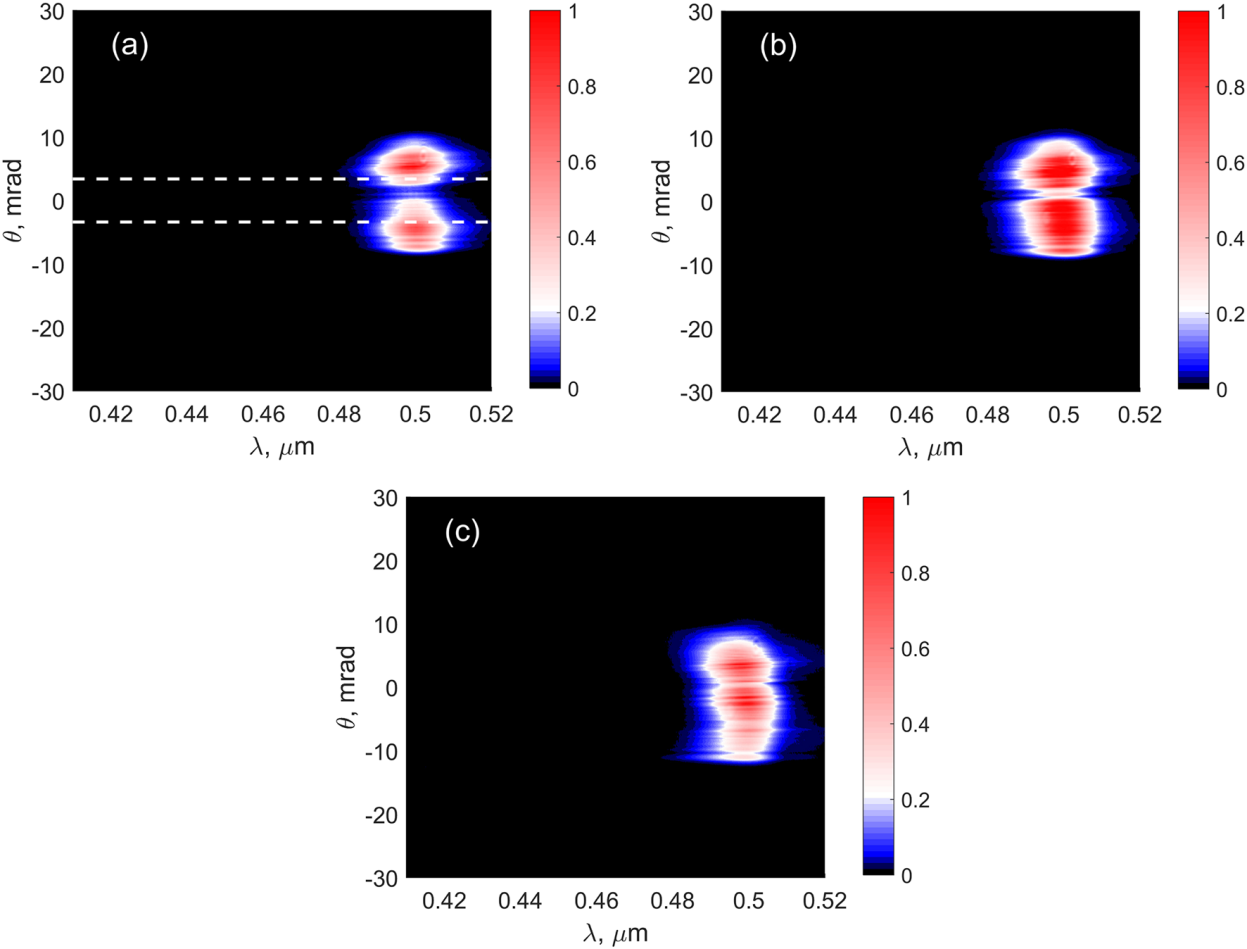
Output TH spectra obtained in experiment for $f=50$ cm for the input energies (mJ): 0.23 (**a**), 0.37 (**b**), 0.53 (**c**). Dashed lines in (**a**) show the longitudinal phase matching analogously to Fig. 3.

**Figure 6:**
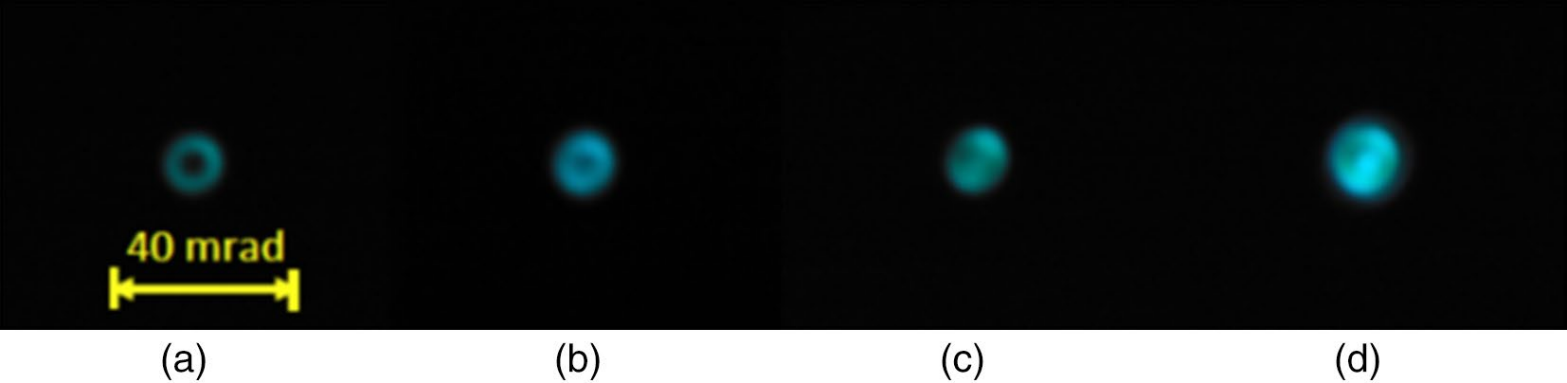
Patterns of TH radiation obtained experimentally, namely displayed on a screen placed beyond the focal plane of the focusing lens. Input energy (mJ): 0.14 (**a**), 0.37 (**b**), 0.59 (**c**), 0.72 (**d**). $f=50$ cm.

Equations (1,2) and (13) were simulated assuming the cylindrical symmetry of the problem, thus the fast Hankel transform for the space-domain was utilized^31^. For the integration, we used the Euler numerical scheme. For the initial conditions, FH at $\lambda _{10}=1.5$ $\upmu$m was assumed, with $r_0=5$ mm and $\tau =30$ fs. In the MPI model, the space window $(0, ~2]\times r_0$ was taken, with a grid consisting of 1500 points, as well as the temporal window $[-7\tau ,~7\tau )$, with a uniform grid containing 128 points. For the YI model, the same spatial grid, and a temporal uniform grid containing 512 points spanning the temporal window $[-4\tau ,~4\tau )$ were used.

**Figure 7:**
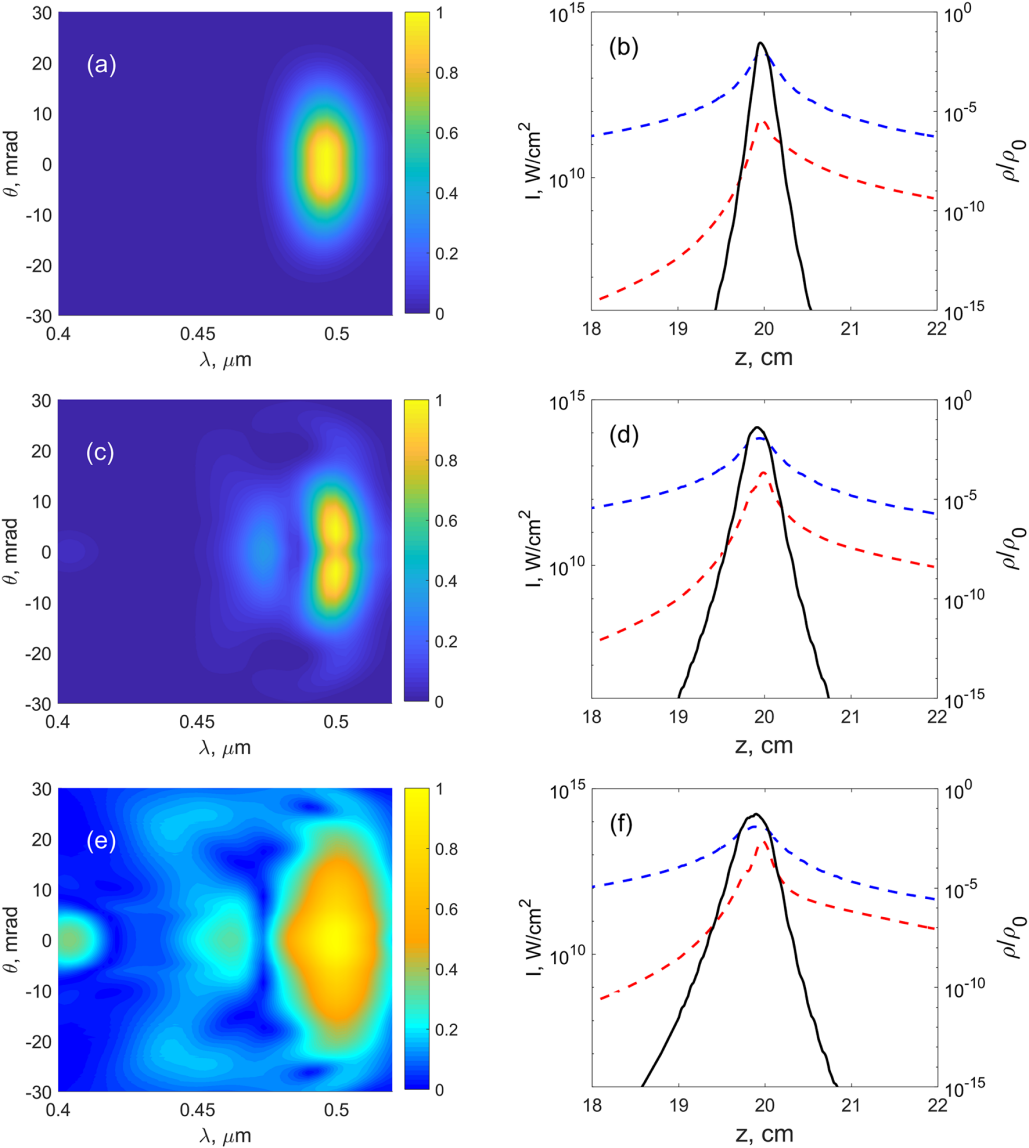
Numerical results for the YI model. (**a**,**c**,**e**) Output TH spectra. (**b**,**d**,**f**) Dependencies of the FH (blue dashed line) and TH (red dashed line) intensities as well as plasma density (black solid line) on the propagation distance. $f=20$ cm, $I_0$ ($10^{10}$ W/cm$^2$): 0.175 (**a**,**b**), 0.525 (**c**,**d**), 1.05 (**e**,**f**). In (**e**), square root of the spectral intensity is depicted.

**Figure 8:**
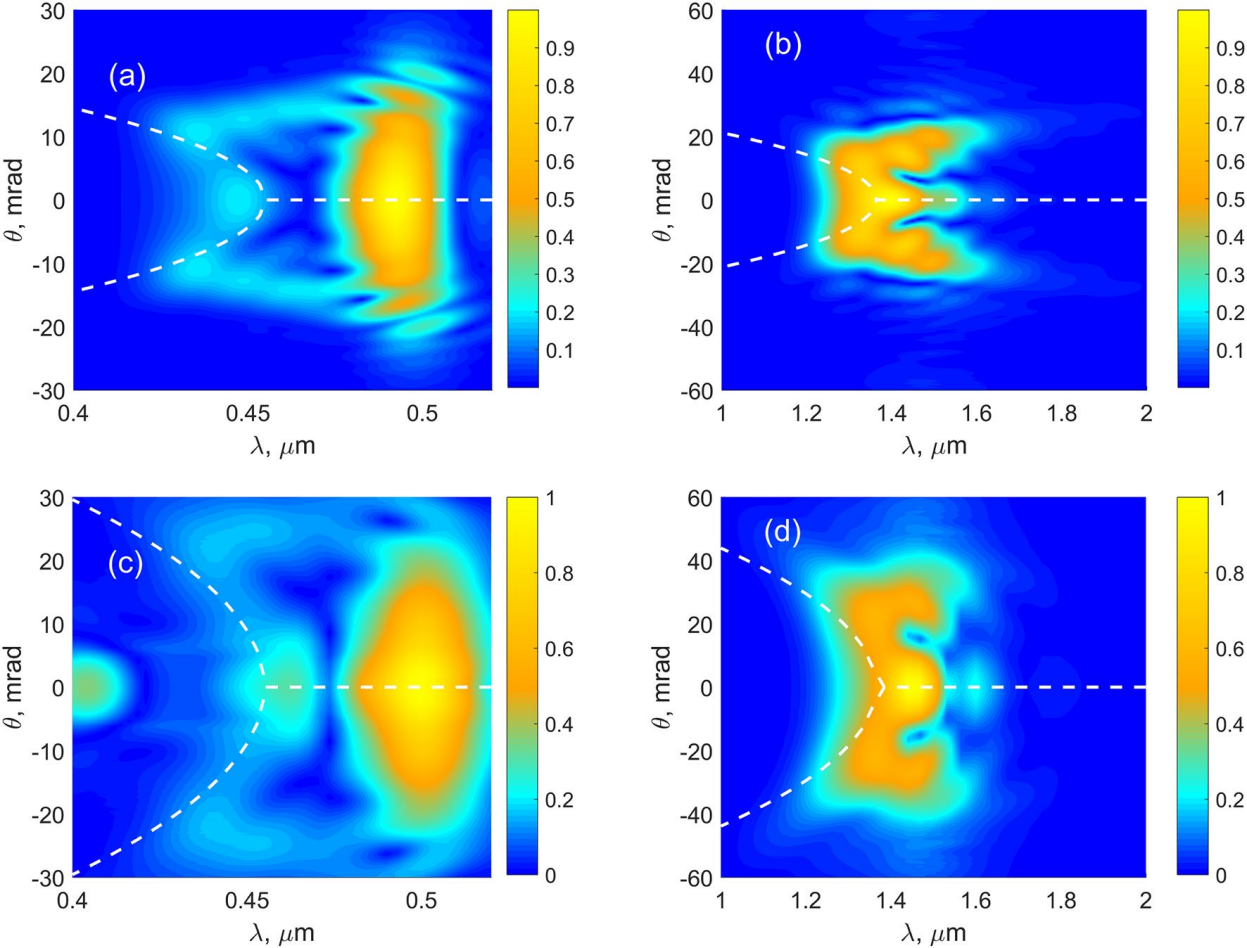
Evidence of X-waves in TH (**a**,**c**) and FH (**b**,**d**) spectra obtained by the MPI model (**a**,**b**) and YI model (**c**,**d**). $f=20$ cm, $I_0$ ($\times 10^{10}$ W/cm$^2$): 1.4 (**a**,**b**), 1.05 (**c**,**d**). Square root of the intensity is shown for better visibility. Dashed lines show dispersion according Eq. (20), with the X-wave group velocity: $3.0017\times 10^{8}$ m/s (MPI model) and $3.01\times 10^{8}$ m/s (YI model). The central fundamental wavelength of the X-waves is $0.455\times 3$ $\upmu$m.

**Figure 9:**
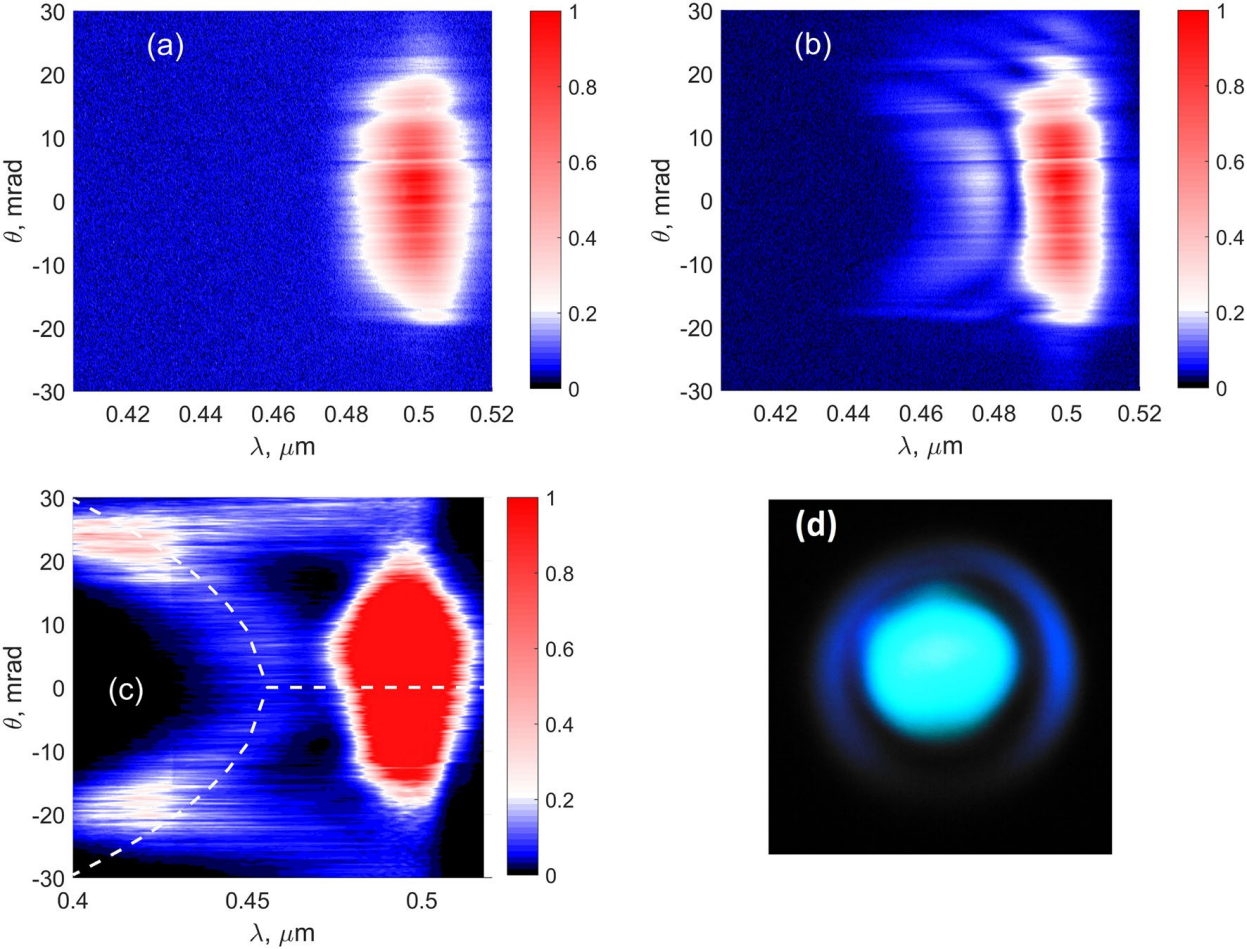
Experimental results. (**a**–**c**) Output TH spectra, $f = 20$ cm, input energy (mJ): 0.11 (**a**), 0.41 (**b**), 0.77 (**c**). Square root of the spectral intensity is taken in (**a**,**b**). Dashed lines in (c) denote the same dispersion curve as in Fig. 8c. (**d**) Typical far-field pattern of output TH beam registered under the same conditions as in (**c**): $f = 20$ cm, input energy 0.77 mJ.

Two values of the focal length were examined: $f=50$ cm (Figs. 3, 4, 5, 6) and $f=20$ cm (Figs. 7, 8, 9). At the larger value of *f* and lower input energy, two maxima in the TH frequency-angular spectrum are observed, see Fig. 3a. These maxima mean the appearance of the one-color ring in the TH angular spectrum. The radius of the ring was found from the longitudinal phase-matching condition ($k_{30}\cos \theta _p-3k_{10}=0$) and is equal to $\theta _p=3.4$ mrad^22,32^ (see dashed lines in Fig. 3a). In this case, the plasma density is low and the intensity clamping is not yet achieved. From the curves in Fig. 3b we see that after passing the focus, the peak intensity of the TH wave (red dashed line) decreases faster than the intensity of the FH wave (blue dashed line) since the ring is formed in the TH spatial profile and consequently the central spot vanishes. At the focus, the TH beam obeys the central spot and its width is smaller than the width of the FH beam.

For the intensities shown in Fig. 3 both MPI and YI models give the same result. This is not surprising, since the peak intensity approaches $10^{11}$ W/cm$^2$ in this case, that is, we are clearly in the multiphoton regime, where both MPI model and YI models being valid. The Keldysh parameter $\gamma$ is equal to 1 at intensity around $3\times 10^{13}$ W/cm$^2$ for the fundamental harmonic, so we expect that as we approach this intensity, MPI and YI models start to deviate, and the tunnel ionization which is not accounted in the MPI model, starts to play a role.

When the input energy increases, the ring merges into a single central spot as seen in Fig. 4a–d, which is observed for both models, although for somewhat different input intensities. In the MPI model, the plasma density becomes saturated at high focal intensity, Fig. 4e, whereas the saturation in the YI model is not so prominent (compare black solid lines in Fig. 4e,f). As a result, the intensity clamping is observed in the MPI model. After passing the focus, the peak intensity of the TH beam falls with the same speed as the peak intensity of the FH beam, compare blue and red dashed lines in Fig. 4e,f. The experimentally recorded spectra of the TH show the same evolution from the ring-shaped spectrum to the central spot, as seen in Fig. 5. In Fig. 6, the experimental TH patterns are shown, which show also the merging. This merging of the ring into a central spot may be attributed to cross- and self-phase modulations of the TH wave. To check this, we repeated the simulation of YI model without the $\chi ^{(3)}$ term and the ring structure did not disappear (data not shown). We also checked the stability of the results with respect to the change of $\Gamma$ and input intensity $I_0$, e.g. we repeated the simulations first with 10 times larger $\Gamma _{1N}$ and second with $I_0$ increased by 1 percent. In both cases, the results are undistinguishable from the initial data.

**Figure 10:**
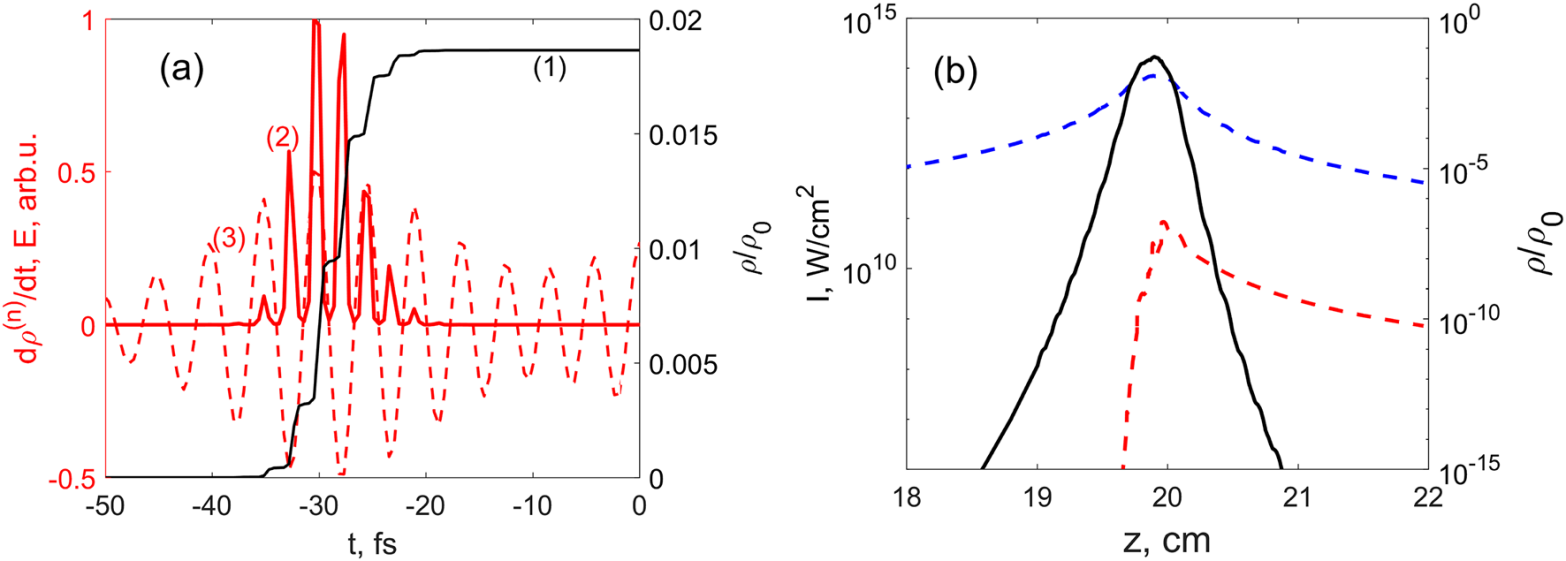
(**a**) Ionization dynamics for the YI model at the beam center for the simulation shown in Fig. 8c,d. Fast oscillations in the plasma density (1, black solid line) and its rate (2, red solid line), sub-cycle in respect to the electric field *E*(*t*) (3, red dashed line) are visible. (**b**) The same as in Fig. 7f, simulated without $\chi ^{(3)}$ term. The influence of Brunel harmonics is much smaller than the one from the $\chi ^{(3)}$ nonlinearity.

At the smaller focal length, $f=20$ cm, larger peak intensity can be achieved. The TH spectra become noticeably blue-shifted, see Fig. 7a,c,e. In these figures, only the results for the YI model are shown and the comparison with the MPI model will be provided below. The intensity clamping is observed, e.g. in Fig. 7b,d,f at the maximum FH intensity around $7\times 10^{13}$ W/cm$^2$ for all three $I_0$ values. From the dashed lines in Fig. 7b,d,f we see that TH and FH intensities of the beams follow the same trend. We think that the signal at 400 nm (Fig. 7e) is a result of the cascaded process of mixing of TH at 500 nm and FH at 1.6 $\upmu$m. This wavelength component in FH appears due to nonlinear broadening of the pump (see Fig. 8b,d).

In Fig. 8, one can see that the locked FH and TH X-waves travelling at the same group velocity are formed^22^. This result is obtained in both YI- and MPI-models, but the group velocities are different in both models (cf. Fig. 8a,b and Fig. 8c,d). In Fig. 8, we also depicted the X-wave dispersion curves by the dashed lines. The dispersion law is given by:

20 $$\begin{aligned} k_{1,3}(\omega )\cos \theta =\frac{\omega -\omega _0}{V}+k_{1,30}, \end{aligned}$$

where $k_{1,3}$ is the frequency dependent wavenumber and $k_{1,30}$ was calculated at the central wavelengths of $0.455\times 3$ $\mu$m (FH) and 0.455 $\upmu$m (TH). *V* is the group velocity of the X-wave. The group velocity *V* was varied by the step of $10^4$ m/s to get the best coincidence with the numerically calculated contours. The resulting $\theta (\lambda )$ following from Eq. (20) is depicted in Fig. 8 by dashed lines. Good coincidence with our experiment and also^22^ is observed in the case of the YI model. In particular, we found the same group velocity $V=3.01\times 10^8$ m/s as in^22^. In contrast, the group velocity obtained from the MPI model deviates from this value: $V=3.0017\times 10^{8}$ m/s. This fact is an evidence of the influence of tunnel ionization on the X-wave formation.

The experimental TH spectra are shown in Fig. 9. Comparison with the numerical results of the YI model in Fig. 7 shows a good agreement. To facilitate such comparison, the dispersion curve resulted from the YI model is shown on the experimental graph (Fig. 9c) by a dashed line. We note that in the theoretical spectra, the angle was calculated as $\theta =k_{\perp }/k_{30}$, where $k_{\perp }=\sqrt{\beta _x^2+\beta _y^2}$ is the transverse projection of the wavevector. The formula is only approximately correct. So, the experimental angle $\theta$ is slightly smaller than the theoretical one at the wavelengths shorter than $\lambda _{30}=500$ nm.

In order to examine the influence of tunnel ionization we provide an exemplary picture of the free electron density (curve 1) and ionization rate (curve 2) shown in Fig. 10a, at the center of the beam for the case of Fig. 8c,d. Here, the oscillations are prominent and the sub-cycle tunnel dynamics are dominant comparing to the multiphoton ones. The question may arise if the Brunel harmonics provided by plasma dominate also the third harmonic generated by $\chi ^{(3)}$ nonlinear term? To answer this question we switched off the $\chi ^{(3)}$ term in the YI model and repeated the simulation for the parameters of Fig. 7f. The results are presented in Fig. 10b. Comparison of these two figures shows that the TH intensity of the plasma radiation is by around two orders lower than the full TH intensity.

## Discussion and conclusions

In conclusion, evolution of the third-harmonic spectrum from the ring-type to locked X-waves by increasing the focal pump intensity was analyzed both theoretically and experimentally. For the theoretical consideration, two models were used, the MPI model based on multiphoton ionization and YI model, working in multiphoton, tunnel as well as in intermediate ranges. We observed several distinct stages of evolution of TH beam. At low intensities, TH forms a ring, which radius is determined by the longitudinal phase-matching. This ring merges into a single central spot at higher intensities due to self- and cross-phase modulations. At even higher intensities, locked X-waves in the third- and fundamental harmonics are generated and co-propagate with the same group velocity. We found two different values of the group velocity of the X-waves: one from the MPI model and another one from the YI model. We observe that the latter value coincides with the experimental one and it is in agreement with the previous experimental work^22^, in contrast to the value given by the MPI model.

We also observed that the MPI model gives rise to intensity clamping at smaller values of the peak intensity than the YI model due to overestimation of the plasma density in MPI model. The spectral-angular X-wave distributions predicted by YI theory are much closer to the experiment than the ones predicted by the MPI model. Since the YI model allows for both slow multiphoton and fast sub-cycle tunnel dynamics, we also checked that for intensities, corresponding to formation of X-waves, the sub-cycle tunnel dynamics clearly dominates the multiphoton one.

These arguments allow us to conclude that the tunnel ionization plays the critical role in the TH X-wave formation in filaments, at least in air for $1.5 \,\upmu \hbox {m}$ wavelength. Because of onset of tunnel ionization, one could also expect that at least part of TH is produced by the plasma-induced nonlinearity and not by $\chi ^{(3)}$-based one. Nevertheless, the results presented here demonstrate, that $\chi ^{(3)}$ nonlinearity remains still the dominant TH source.
